# The Physician’s Visiting List

**Published:** 1855-12

**Authors:** 


Art. VIII.—*The Physician's Visiting List, Diary, and Book of Prescriptions, for* 1856. Philad.: Lindsay & Blakiston.

Having once used it one cannot do without it. For sale in this city by Maxwell & Co. D. W. Y.

